# Event and Comment

**Published:** 1920-07

**Authors:** 


### DENTAL REGISTER

THE MAGAZINE OF DENTISTRY

Vol. LXXIV | JULY, 1920 | No. 7

### EVENT AND COMMENT

**University of Rochester** Gifts of nearly ten million dollars to George Eastman and the Rockefeller General Education Board make possible the establishment of the medical and dental departments of the University of Rochester on a permanent and a high-grade basis of efficiency and standardization. The inspiration for this action is largely the result of the successful conduct of the work done by the Rochester Dental Dispensary. The work accomplished by the dentists in their efforts to take care of the teeth of the children of Rochester in years gone by brought about the establishment of that magnificent building which is doing such a great work to providing for the future dental needs as well as the general health of the poor children in that city. The idea is now to broaden this effort to secure better physical health for the entire body as well as for the teeth. The establishment of a medical and dental faculty of the grade proposed, working in close and harmonious co-operation and with ideal environments, is destined to accentuate the need for humanitarian efforts of the finest type and with the most enduring and satisfactory results. Incidentally this is going to be one of the greatest experiments ever made in scientific education. If it is possible to demonstrate that the world can be redeemed in large measure by creating better physical conditions, it will certainly make the world happier, because healthier.

We are accustomed to stress efficiency in various departments of business and other executive affairs, and the world has apparently benefitted by more exact and systematic organization. It now seems that our own profession is lining up for larger opportunities. The demand is that we shall keep pace with the great developments of this most important matter of good health in general as we have already emphasized it in some particulars. The ideal seems to be to co-operate dental and medical instruction in an effort to secure an accurate and efficient technical education which will best serve the needs of people who require health of the body as well as sanitary and prophylactic instruction.

There will always be a demand for technical medical endeavor, which should be of the highest type obtainable. While specialization tends to efficiency, a strong and broad fundamental training is essential for the greatest real advancement. Therefore, to obtain the best results in efficiency we should gain tremendously by co-ordinating our efforts in education with special training, as is proposed to be done in a university completely organized and supported upon these definite fundamental ideals. While this university has definite aims and purposes, it is not to be a one-idea institution; it will expand naturally and competently to the extent it is wisely managed. The philanthropic and segacious conduct of Mr. Eastman's previous endeavors assures the future of this great undertaking.

Literature, science, and the arts are being happily combined for ideal training in the useful arts and sciences, and the University of Rochester steps at once into the front rank of great and useful universities.

